# Jefferson Medical College

**Published:** 1830

**Authors:** Samuel McClellen

**Affiliations:** Dean. Philadelphia


*Jefferson Medical College.*—The *Jefferson Medical College* was instituted in Philadelphia, in 1825, and, during its first session, was endowed by an act of the Legislature of Pennsylvania, with power to confer degrees in Medicine, and with all the privileges and prerogatives of similar Institutions, in our own country and in Europe.

Since its foundation nearly 600 students have attended the respective courses of Lectures, and of this number, 145 from various sections of the United States, the Canadas, West Indies, and Europe, have been admitted to the Degree of Doctor of Medicine.

The Sixth Session of the College will commence on the first Monday of November next, and, it is believed, under the most favorable auspices; every obstacle to its future advancement having been removed, and such measures adopted by the Board of Trustees, as will give a new impulse to the exertions of the faculty. Among these measures, it is thought proper to specify particularly, the appointment of Professor Drake, of Cincinnati, to the chair of the Practice of Medicine. His reputation as a lecturer is acknowledged, while his practical acquaintance with the diseases most prevalent in the Southern and Western sections of our country, in which a majority of the graduates in medicine may be expected to locate themselves, must greatly enhance the value of his instruction.

The additions which have been made to the Anatomical Cabinet, with the facilities afforded for dissection and demonstration, are such as will bear comparison with those of the oldest Medical Schools on this side of the Atlantic.

In all other respects it is confidently believed, it is not surpassed by any of its sister institutions, with *all* of which, as far as is known, it is placed on a footing of perfect equality—a course of Lectures in one, being held equivalent to a course on the same branches, in every other.

The Alms House and Hospital of the city afford ample opportuuities to the student of acquiring clinical instruction, and hours are appropriated to this purpose. An Infirmary for diseases of the eye is also connected with the College, under the direction of the Professor of Surgery, who will operate in the presence of the class, where this is practicable.

The following is the organization of the Medical Faculty—viz:

*Anatomy*—By SAMUEL McCLELLEN, M. D.

*Materia Medica and Obstetrics*—By JOHN EBERLE, M. D.

*Chemistry*—By JACOB GREEN, M. D.

*Theory and Practice of Medicine*—By DANIEL DRAKE, M. D.

*Surgery*—By GEORGE McLELLEN, M. D.

*Institutes of Medicine, Medical Jurisprudence, and the diseases of women and children*—By B. RUSH RHEES, M. D.

The fee for attendance on each course is 15 dollars, or 90 dollars for all the courses. The graduation fee is 15 dollars, and no other charge is made either on matriculation, or subsequently.

The whole expense is therefore less than 200 dollars for the two full courses, required by law to entitle the student to graduation. One of these, at least must be attended in this institution; and it is further required, that the candidate shall have studied three years (inclusive of the terms of attendance on the lectures) under the direction of a respectable practitioner of medicine,—that he shall be twenty one years of age—and having presented a thesis on some medical subject, shall give satisfactory evidence of his qualifications on an examination by the Faculty, which takes place immediately after the close of each session.

The annual commencement for conferring the degrees, will not be delayed beyond the time requisite to complete the examination, that no unnecessary expense may be incurred by the student for board. This may be procured in Philadelphia, at the rate of from two dollars and fifty cents to four dollars, in very respectable houses, to which the Janitor will furnish references, without charge.

Ten students are by law admitted gratuitously, each session, (the sum of twenty dollars only being paid by each to meet the current expenses of the institution.) Application for the benefit of this provision, and all other communications to the Faculty, must be addressed to Samnel McClellen, M. D. Dean of the Faculty, at the Jefferson Medical College, 10th, near Walnut street, Philadelphia. By order of the Faculty.

SAMUEL McCLELLEN, *Dean.*

*Philadelphia, March,* 1830.

